# Environmental factors determining the distribution patterns of invasive *Raphidiopsis raciborskii* and *R. mediterranea* in central east Europe

**DOI:** 10.3389/fmicb.2025.1533716

**Published:** 2025-05-07

**Authors:** Mikołaj Kokociński, Caio Graco-Roza, Iwona Jasser, Jūratė Karosienė, Jūratė Kasperovičienė, Justyna Kobos, Judita Koreivienė, Joanna Mankiewicz-Boczek, Janne Soininen, Agnieszka Szczurowska

**Affiliations:** ^1^Department of Hydrobiology, Institute of Environmental Biology, Adam Mickiewicz University, Poznań, Poland; ^2^Department of Geosciences and Geography, University of Helsinki, Helsinki, Finland; ^3^Lammi Biological Station, University of Helsinki, Lammi, Finland; ^4^Faculty of Biology, Institute of Environmental Biology, University of Warsaw, Warsaw, Poland; ^5^Laboratory of Algology and Microbial Ecology, State Scientific Research Institute Nature Research Centre, Vilnius, Lithuania; ^6^Division of Marine Biotechnology, Faculty of Oceanography and Geography, University of ń, ń, Gdynia; ^7^European Regional Centre for Ecohydrology of the Polish Academy of Sciences, Łódź, Poland; ^8^Department of Botany and Plant Physiology, University of Life Sciences in Lublin, Lublin, Poland

**Keywords:** cyanobacteria expansion, freshwater lakes, nutrients, non-native cyanobacteria, temperature

## Abstract

**Objective:**

In recent decades, the invasive cyanobacteria *Raphidiopsis raciborskii* and *Raphidiopsis mediterranea* have expanded their distribution globally, particularly in temperate regions. Understanding the ecological drivers of *Raphidiopsis* distribution is imperative to addressing the challenges associated with these species. Here, we aimed to characterize the distribution and biomass of *R. raciborskii* and *R. mediterranea* across 112 lakes in Poland and Lithuania in relation to local and regional factors.

**Research design and methods:**

Integrated water samples were collected from 102 Polish and 10 Lithuanian lakes from different regions for phytoplankton and chemical analyses. The lakes varied in surface area, and exhibited diverse mixing regimes, trophic states, and morphometries. Phytoplankton was identified and quantified using a Fuchs-Rosenthal or Nageotte chamber. Additionally, we characterized the degree of human pressures the climatic constraints experienced by each lake.

**Results:**

*R. raciborskii* occurrence has increased in eastern regions of Poland but biomass is relatively low compared to western Poland, likely due to lower air temperatures and nutrient concentrations, especially phosphorus. In contrast, *R. mediterranea* only occurred in a small number of lakes in Poland, and in a single lake in Lithuania, with no relation to measured local and regional variables.

**Conclusions:**

Our study shows contrasting patterns in the distribution of two invasive cyanobacteria species in Europe, highlighting the importance of climate and nutrients to the distribution of *R. raciborskii*, the most widespread species, and providing relevant information for decision making and conservation strategies.

## 1 Introduction

Rising lake water temperatures related to global warming and eutrophication are two major factors enhancing the expansion of invasive cyanobacteria and the development of harmful algae blooms (HABs) (Paerl and Huisman, 2009; Kosten et al., 2011; Lürling et al., 2017). Increasing freshwater productivity due to higher level of nutrients relates strongly to changes in catchment land use (Hupfer and Hilt, 2008). Intensive land use (especially agriculture and urban activities) accelerates nutrient delivery to water bodies, particularly in the areas with poor wastewater treatment (de Jonge and Elliott, 2001). Consequently, biodiversity typically decreases, with harmful changes in ecosystem function. Furthermore, new threats may emerge when toxic cyanobacteria strains dominate and release toxins into the water.

Among the species that often dominate HABs, *Raphidiopsis raciborskii* (Wołoszyńska) Aguilera, Berrendero Gómez, Kástovsky, Echenique and Salerno originates from tropical and subtropical regions, but it has expanded into temperate zones over the last several decades (Padisák, 1997; Sinha et al., 2012; Burford et al., 2016; Aguilera et al., 2018). *R. raciborskii* was recorded in Europe for the first time at Lake Kastoria in Greece (Skuja, 1937), from where colonization of other European countries likely started (Padisák, 1997). In many countries, it is considered an invasive species and a serious threat to native phytoplankton communities, disrupting natural ecosystem processes (Sukenik et al., 2012; Wilk-Woźniak et al., 2016). Recent studies showed increasing occurrence of *R. raciborskii* in both hemispheres with the northernmost (57°N) recorded case in Nero Lake, Russia suggesting it is now a cosmopolitan species (Panou et al., 2018; Sidelev et al., 2020). Previous studies have also indicated that *R. raciborskii*, can become dominant within a relatively short period (Padisák, 1997).

Evidence suggests that *R. raciborskii* exhibits phenotypic plasticity, allowing it to colonize diverse freshwater ecosystems. This adaptability relates to its highly flexible ecotypes, enabling it to cope with environmental changes, including shifts in climate, explaining the successful expansion toward more northern regions (Burford et al., 2016). Studies show that *R. raciborskii* thrives across a wide range of water temperatures and light conditions. Nevertheless, its environmental optima is warmer waters, typically ranging between 29°C and 32°C (Thomas and Litchman, 2016; Xiao et al., 2017; Zheng et al., 2023). Blooms of R. *raciborskii* usually occur when water temperature exceeds 25°C (Recknagel et al., 2014; Jia et al., 2021); however, in Langer See, Germany, *R. raciborskii* reached its highest abundances at 24°C, even when higher temperatures were recorded during the summer seasons (Wiedner et al., 2007; Rücker et al., 2009). In tropical regions, where temperatures typically exceed 20°C, perennial blooms commonly occur (Recknagel et al., 2014; Jia et al., 2021). Despite optima in warmer waters, there are records of viable *R. raciborskii* populations at temperatures as low as 8°C–11°C (Bonilla et al., 2012; Dokulil, 2015).

Light availability also plays an important role in *R. raciborskii* distribution, with evidence of growth at light intensities ranging from 8.5 to hundreds of μmol photons m^−2^ s^−2^ (Briand et al., 2004; Carneiro et al., 2009; Bonilla et al., 2012; Mehnert et al., 2010). However, high water temperature and irradiance levels (above 2,000 μmol photons m^−2^ s^−2^) may inhibit growth and bloom formation (Kehoe et al., 2015). Available data on growth rate response to light intensity originate from different temperatures, suggesting an interactive effect between light and temperature on *R. raciborskii*. The ongoing rise in air temperature and light attenuation in lakes due to anthropogenic climate change may play significant role in further successful spatial expansion of *R. raciborskii*.

In addition to broad preferences for light and temperature, *R. raciborskii* also exhibits high uptake affinity and storage capacity for phosphorus (Antunes et al., 2015), which proves beneficial under conditions of pulsed discharge or fluctuating concentrations of dissolved phosphorus (Marinho et al., 2013; Amaral et al., 2014). As for nitrogen, *R. raciborskii* shows a preference for dissolved inorganic (ammonia and nitrate) and organic (urea) forms, but also can fix N2 nitrogen by terminal heterocytes under nitrogen depletion (Plominsky et al., 2013; Ammar et al., 2014; Burford et al., 2018). The capacity to regulate buoyancy and akinete formation support growth and dominance even under suboptimal environmental conditions (Sukenik et al., 2012). Altogether, these traits reflect multiple strains with differing physiological responses across range of environmental conditions (Pierangelini et al., 2015; Willis et al., 2015; Xiao et al., 2017), resulting in successful range expansion.

In addition to *R. raciborskii*, *Raphidiopsis mediterranea* Skuja has increased occurrence in temperate zones. Like *R. raciborskii*, it was initially described in Lake Kastoria, Greece, suggesting a Mediterranean origin (Kaštovský et al., 2010). *R. mediterranea* occurs worldwide, from tropical and subtropical regions (e.g., Australia, Brazil, China) to temperate regions (e.g., Bulgaria, Czech Republic, Serbia) (Wilk-Woźniak et al., 2016). Notably, continuous populations have been documented in six Lithuanian lakes (Kasperovičienė et al., 2010; Kaštovský et al., 2010). Despite these global reports, our knowledge of its ecological preferences and current distribution remains incomplete. *R. mediterranea* thrives in a variety of aquatic environments, including eutrophic to hypertrophic, shallow, and deep lakes or ponds (Kasperovičienė et al., 2010; Wilk-Woźniak et al., 2016; Aguilera et al., 2018). It exhibits adaptability to a broad temperature range, with growth observed between 0.5°C and 17.9°C (Cronberg, 1973). While some studies indicate year-round growth, *R. mediterranea* has only been documented in the temperate zone, for example in the Lithuanian lakes, during warmer summer months (July–August) when water temperature ranges between 20°C and 25°C (Kasperovičienė et al., 2010). Similarly to *R. raciborskii*, *R. mediterranea* has the potential to coexist with other cyanobacteria during toxic blooms (Watanabe et al., 2003; Namikoshi et al., 2003; Mohamed, 2007).

*R. raciborskii* and *R. mediterranea* are both potential cylindrospermopsin producers (McGregor et al., 2011; de La Cruz et al., 2013), but such production has not yet been reported in Europe. There are also known strains from outside Europe capable of producing neurotoxins, including saxitoxin by *R. raciborskii* (Lorenzi et al., 2016) and anatoxin-a by *R*. *mediterranea* (Namikoshi et al., 2003). Moreover, some European strains of *R. raciborskii* can produce chemical compounds of yet unknown structure that may cause oxidative stress and cytotoxic and neurotoxic effects in human or animal cells (Poniedziałek et al., 2015; Rzymski et al., 2017a; Rzymski et al., 2017b; Falfushynska et al., 2019).

A 2014 study showed widespread occurrence of *R. raciborskii* in lakes impacted by agriculture and urbanization in Western Poland (Kokociński et al., 2017), with *R. raciborskii* recorded in 25 out of the 117 lakes sampled. Notably, this study highlighted an increasing contribution of *R. raciborskii* to the overall phytoplankton biomass in Western Polish lakes and no occurrence of this species in Eastern Poland, including the north-eastern lake districts. R. *raciborskii* was less prevalent in Lithuania, observed in only one lake (Kokociński et al., 2017). In contrast, *R. mediterranea* exhibited limited distribution in Polish lakes compared to Lithuanian water bodies in recent years (Kasperovičienė et al., 2010). Since 2004, *R. mediterranea* has been identified in six out of 18 lakes investigated, contributing up to 15% of the total phytoplankton biomass (Kasperovičienė et al., 2005).

To summarize, it is imperative to deepen our understanding of the distribution and ecological factors influencing the proliferation of *R. raciborskii* and *R. mediterranea*. We therefore aim to (i) characterize the current distribution of *R. raciborskii* and *R. mediterranea* in lakes across Poland and Lithuania, (ii) explore the roles played by local environmental variables, land use and climate in shaping the distribution of *R. raciborskii* and *R. mediterranea*, and (iii) investigate whether the occurrence and biomass of *R. raciborskii* have increased in recent years.

## 2 Materials and methods

### 2.1 Study sites

To examine the environmental factors driving the distribution of *R. raciborskii* and *R. mediterranea* in the temperate zone, we sampled 102 Polish and 10 Lithuanian lakes from different regions (Supplementary Table 1). The lakes varied in surface area, ranging from 5 to 11.4 ha, and exhibited diverse mixing regimes, trophic states, and morphometries. Among the 112 lakes surveyed, 25 were classified as very shallow (max. depth < 5 m), 34 as shallow (5–10 m), and 53 as deep (> 10 m). Additionally, land use showed considerable variation, with Western Poland featuring agricultural and urban catchments, while Eastern Poland and Lithuanian catchments were predominantly characterized by forest land use.

### 2.2 Sample collection

Samples were collected once for each lake during the summer (July–September) 2020. Integrated phytoplankton samples were collected from the water column in polymictic lakes or epilimnion in stratified lakes from one sampling station using bathometer. The phytoplankton samples were preserved with acidified Lugol’s solution with a final concentration of 1% (APHA et al., 2012; Hötzel and Croome, 1999) and stored under cool and dark conditions until analysis.

### 2.3 Phytoplankton analysis

Phytoplankton was identified and quantified using a Fuchs-Rosenthal or Nageotte chamber. Two subsamples were analyzed for each lake. To ensure accuracy, a minimum of 400 cells or filaments were counted, reducing the error to less than 10% (Olenina et al., 2006). Phytoplankton biomass, measured in fresh weight, was determined through cell volumetric analysis using geometric approximation and expressed as a wet weight following Wetzel and Likens (2000).

### 2.4 Physico-chemical analysis

Simultaneously with the collection of phytoplankton samples, water samples were collected for chemical analyses. Total nitrogen (TN), total phosphorus (TP), dissolved nitrogen (DN) and total reactive phosphorus (TRP) concentrations were determined by a spectrophotometric method. For a chlorophyll a (chl a) analysis, 200–500 mL of water was filtered through a GF/C Whatman filter. The concentration was determined spectrophotometrically after extraction with 90% acetone, and calculations were based on Lorenzen’s formula (Wetzel and Likens, 2000). Field measurements included water temperature, pH, and conductivity using a multiparameter probe, while water transparency was measured using a Secchi disk (SD).

### 2.5 Land use and climatic variables

We characterized the degree of human pressures experienced by each lake by estimating the proportion of urban and agricultural environments within a radial buffer of 2 km around the lake centroid. The human-associated land cover classes (two out of 17 landcover classes in total) were obtained from the “Land_Cover_Type_1” raster layer of the “MCD12Q1” data product of NASA’s Moderate Resolution Imaging Spectroradiometer (MODIS) (Friedl and Sulla-Menashe, 2022). In addition, we characterized the climatic constraints of each lake by using measures of the mean annual temperature (Air temperature; °C) and total annual precipitation (Precipitation; mm) from a 1 km radial buffer around each lake centroid. The climatic variables used (two out of 19 variables) were obtained from WorldClim v.2.0 (Fick and Hijmans, 2017).

## 3 Data analysis

We analyzed data using R version 4.2.0 (R Core Team, 2022) and the packages stats v.4.2.0 (R Core Team, 2022), “nlme” v.3.1–157 (Pinheiro and Bates, 2000; Pinheiro and Bates, 2022), and the “tidyverse” suite of packages (Wickham et al., 2019).

To determine the role of local environmental variables, land use and climate in the distribution of *R. raciborskii* and *R. mediterranea* we modeled the relationship between *R. raciborskii* biomass and distribution as a function of area (Log km^2^), mean depth (Log m), conductivity (log μS cm^–1^), Secchi depth (log m), total phosphorus (log mg l^−1^), total reactive phosphorus (log mg l^−1^), total nitrogen (log mg l^−1^), air and water temperature (°C), precipitation (mm), proportion of urban area (% in 2 km radial buffer), and proportion of agricultural area (% in 2 km radial buffer) using a two-step modeling approach. First, we modeled the local and broad scale factors explaining *R. raciborskii* presence. We included the distribution of *R. raciborskii* across lakes as a response variable to be explained by the set of environmental factors using a general linear model (GLM) following a binomial distribution of the errors fitted with the package “stats.” Second, we modeled the effects of local and broad scale factors on *R. raciborskii* biomass using generalized least squares regression (GLS) fitted with the package “nlme.” To account for spatial autocorrelation, we introduced an exponential correlation structure on the geographic coordinates (longitude and latitude) of each lake. We validated our models using a suite of diagnostic tests via the check-model function from the performance package (version 0.12.3) in R. Specifically, we evaluated residual normality (using Q–Q plots), homoscedasticity (by plotting residuals versus fitted values), and the influence of individual observations (using leverage and Cook’s distance). In addition, we computed Variance Inflation Factors to assess multicollinearity. Collectively, these diagnostics confirmed that our models satisfied the necessary assumptions for reliable inference (Zuur and Ieno, 2016). Model performance was moderate, explaining 0.26 and 0.30 of the variation in biomass and distribution of *R. raciborskii*, respectively. In addition, we used Generalized Additive Model to test for potential non-linear relationships between predictor variables and the biomass and distribution of *R. raciborskii*. Analysis did not show any non-linear relationships and did not explain more variability in species occurrence and thus results are not shown here.

We note that *R. mediterranea* was identified in only seven lakes, and therefore provided insufficient sample size for modeling the occurrence and biomass of this species.

## 4 Results and discussion

### 4.1 Distribution pattern of *R. raciborskii* and *R. mediterranea*

**FIGURE 1:**
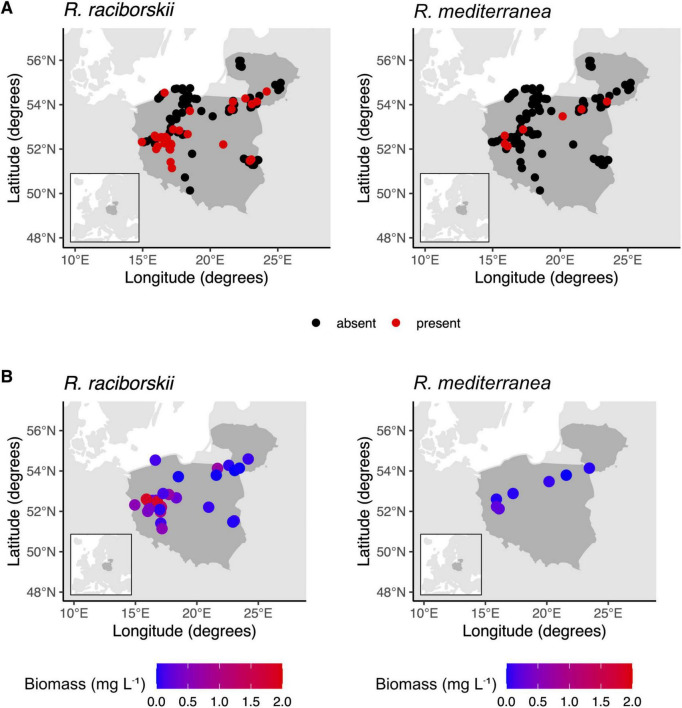
The occurrence **(A)** and biomass **(B)** of *Raphidiopsis raciborskii* and *Raphidiopsis mediterranea* in Polish and Lithuanian lakes.

Our study contributes to the understanding of *R. raciborskii* and *R. mediterranea* distribution in Central East Europe. We identified *R. raciborskii* in 31 out of 112 surveyed lakes (Figure 1A) and occurrence was related mainly to decreasing TRP and increasing TN (Table 1). Kokociński et al. (2017) showed that since 2006, *R. raciborskii* biomass has consistently increased, although it is not a dominant species, in contrast to southern European regions, where *R. raciborskii* frequently dominates the phytoplankton community (Bolius et al., 2017). Our research identifies new occurrences of *R. raciborskii* in lakes within the Warmia and Masuria district and other regions of eastern Poland since 2007, when this species was only reported in two lakes (Jakubowska et al., 2013).

**TABLE 1:** Modeling the distribution of the species *Raphidiopsis raciborskii.*

	Log biomass Generalized least squares Gaussian (R^2^ = 0.260)					Presence/absence Generalized linear model Binomial (Tjur’s R^2^: 0.303)				
**Variable**	**Estimate**	**Standard Error**	**t-statistic**	***P*-value**	**VIF^1^**	**OR^2^**	**Standard Error**	**z-value**	***P*-value**	**VIF^1^**
Intercept	–1.25	0.63	–2.00	**0.05**		0.02	6.16	–0.68	0.50	
Log area (km^2^)	–0.03	0.02	–1.75	0.08	1.37	0.88	0.19	–0.69	0.49	1.42
Log mean depth (m)	0.04	0.07	0.51	0.61	2.07	1.25	0.72	0.31	0.76	2.00
Log conductivity	0.12	0.07	1.80	0.07	1.73	1.90	0.61	1.06	0.29	1.58
Log visibility	–0.04	0.09	–0.52	0.60	2.19	0.51	1.00	–0.68	0.50	2.25
Log total phosphorus	–0.38	0.41	–0.91	0.36	1.62	4.20	3.24	0.44	0.66	1.48
Log total reactive phosphorus	–1.49	0.98	–1.52	0.13	1.66	0.00	10.66	–2.06	**0.04**	1.70
Log total nitrogen	0.20	0.11	1.86	0.07	2.11	8.61	0.99	2.17	**0.03**	1.91
Air temperature (°C)	0.10	0.05	2.09	**0.04**	2.21	1.38	0.46	0.70	0.48	2.14
Precipitation (mm)	0.00	0.00	0.09	0.93	1.89	0.99	0.01	–1.00	0.32	1.95
% Urban areas (km^2^)	0.14	0.21	0.68	0.50	1.08	10.75	1.98	1.20	0.23	1.05
% Agricultural areas (km^2^)	–0.01	0.10	–0.10	0.92	1.34	1.80	0.91	0.65	0.52	1.27

^1^Variance inflation factor.

^2^Odds ratio. Significant variables at *P* < 0.05 are marked in bold.

We did not find any new occurrences of either species in Lithuanian lakes. Despite expanding occurrence of *R. raciborskii* to the east, our data show that biomass differed between regions. *R. raciborskii* biomass ranged from 0.02 to 5.90 mg L^−1^ and was much higher in lakes in Western Poland (on average 1.08 mg L^−1^) than Eastern Poland (on average 0.14 mg L^−1^) (Figure 1B and Supplementary Table 1), mainly driven by increased air temperature (Table 1). Contribution to total phytoplankton biomass ranged from 0.1% to 31% and was also higher in Western Poland (on average 7.4%) than Eastern Poland (on average 0.9%). Moreover, lower occurrence and contribution to phytoplankton biomass in Eastern Poland, along with the sole occurrence in Lithuania, suggest environmental barriers inhibiting colonization toward eastern continental zone of Europe.

Compared with *R. raciborskii*, *R. mediterranea* was recorded much less frequently (Figure 1A). We identified individuals in seven of 112 lakes, too small of a sample size to associate these occurrences with possible drivers. Still, we report three new occurrences of *R. mediterranea* in lakes located in the eastern part of the country (Figure 1A). In Poland, it occurred in different regions without any clear distribution pattern. Biomass and relative contribution of *R. mediterranea* to total phytoplankton biomass were also consistently low, ranging from 0.004 to 0.02 mg L^−1^ (Supplementary Table 1) and 0.10%–2.6%, respectively, aligning with prior reports that underscore limited range expansion (Wilk-Woźniak and Najberek, 2013). In Lithuania, the species was found in only one lake, diverging from the findings of previous years when relatively high biomass was observed in several lakes (Kasperovičienė et al., 2010). This observation shows that, akin to other non-native species such as *Chrysosporum bergii*, *Sphaerospermopsis aphanizomenoides*, and *Cuspidothrix issatchenkoi*, *R. mediterranea* is expanding toward the temperate zone. However, occurrence and contribution to total phytoplankton biomass vary notably across years.

We acknowledge that our sampling protocol may miss some of the species and thus some false absences may appear, but importantly, our sampling effort was the same for all lakes. Moreover, the sampling of large number (117) of lakes should alleviate the problems related to potential false absence. Furthermore, as temporal trends maybe important in plankton, we collected all samples in the middle of vegetative season to further reduce the potential false absences. To sum up, we think that our sampling design comprising large number of lakes is sufficient to provide reliable overview of the species regional distribution and biomass.

### 4.2 Environmental factors driving the abundance and occurrence of *R. raciborskii* and *R. mediterranea*

Among the possible factors influencing the abundance and occurrence of *R. raciborskii*, air temperature, TRP and TN were significant (Table 1). Specifically, air temperature exhibited a positive association with *R. raciborskii* biomass, while nutrient concentrations were related to occurrence. Notably, our findings contrast with many earlier studies emphasizing the important role played by water temperature, which impacts *R. raciborskii* growth and bloom formation (Soares et al., 2012; Recknagel et al., 2014).

The observed positive relationship between *R. raciborskii* biomass and air temperature aligns with a previous study in the same region (Kokociński et al., 2017), highlighting the pivotal role of climate on distribution. Our findings reveal a consistent longitudinal pattern of *R. raciborskii* distribution and biomass in Poland and Lithuania, extending from the west to the east. This pattern can be attributed to the influence of warmer air masses from the Atlantic Ocean, leading to higher temperatures in Western Poland, in contrast to Eastern Poland, where colder air masses from Eurasian landmass prevail (Błażejczyk, 2006). Furthermore, western lakes experience earlier warming in the spring and prolonged warmth in the autumn, resulting in an extended vegetative season compared to lakes in eastern Poland and Lithuania.

Regarding nutrients, we found a negative relationship between orthophosphates and the probability of *R. raciborskii* occurrence. This finding aligns with expectations, considering that *R. raciborskii* employs multiple strategies to thrive under diverse phosphorus conditions (Bonilla et al., 2012; Wu et al., 2022). Notably, it exhibits a particularly high affinity for dissolved inorganic phosphorus (Isvánovics et al., 2000; Wu et al., 2009, 2012) and possesses a substantial phosphorus storage capacity (Posselt et al., 2009; Willis et al., 2017, 2019). Due to this storage capacity, the pulsed addition of dissolved phosphorus is deemed more favorable for growth than constant phosphorus input (Posselt et al., 2009; Amaral et al., 2014). Collectively, these adaptive strategies allow *R. raciborskii* to successfully compete with native cyanobacteria such as *Microcystis aeruginosa* and *Aphanizomenon flos-aquae* (Wu et al., 2009). Our results provide further evidence that *R. raciborskii* has high capability to inhabit and sustain abundant populations in new lakes even under conditions of depleted dissolved phosphorus provided that other environmental conditions are suitable.

Our study also showed that *R. raciborskii* is more likely to be found in lakes with higher TN concentrations. This agrees with previous findings for the same region, where TN played a pivotal role in *R. raciborskii* distribution (Kokociński et al., 2017). While *R. raciborskii* can fix atmospheric nitrogen to thrive under low dissolved nitrogen conditions, it still exhibits preference for certain nitrogen forms (Burford et al., 2006; Ammar et al., 2014). For instance, ammonium nitrogen increased *R. raciborskii* growth rate, allowing it to outcompete native *Planktothrix agardhii* populations (Ammar et al., 2014). Nitrogen concentrations as low as 0.50 mg L^−1^, however, may limit *R. raciborskii* growth (Dai et al., 2015).

Note that we lack data for zooplankton in the study lakes. We acknowledge that the inclusion of zooplankton data (e.g., number of individuals) might have increased somewhat the explained variability by the species distribution model. We think, however, that this study based on abiotic variables only still contribute significantly to our understanding of *R. raciborskii* expansion.

*R. mediterranea* is considered a taxonomically close species or even a non-heterocytous form of *R. raciborskii* (Moustaka-Gouni et al., 2009). However, Aguilera et al. (2018) observed stable native populations of *R. mediterranea* without heterocytes and found no differentiation of heterocytes in isolated strains exposed to nitrogen starvation, suggesting that *R. mediterranea* is likely a distinct species. In this study, *R. mediterranea* was detected in seven water bodies in Poland, and both *Raphidiopsis* species co-occurred in five lakes. However, in most lakes, *R. mediterranea* biomass was 2–200 times lower than *R. raciborskii* biomass. The results also show that water transparency was lower and total and reactive phosphorus concentrations were higher in lakes where *R. mediterranea* or both species were present (mean Secchi depth 0.74 ± 0.55 m; mean TP 0.180 ± 0.138 mg L^–1^; mean TRP 0.063 ± 0.040 mg L^–1^) compared to lakes containing only *R. raciborskii* (mean Secchi depth 1.40 ± 1.54 m; mean TP 0.121 ± 0.105 mg L^–1^; mean TRP 0.040 ± 0.039 mg L^–1^). Similarly, Aguilera et al. (2019) demonstrated that water temperature, light availability and phosphorus are key factors influencing the proliferation of *R. mediterranea* isolates and competition with other cyanobacteria species. Previously, *R. mediterranea* was detected in six Lithuanian lakes (Kasperovičienė et al., 2005, 2010; Kasperovičienė, 2007) and correlated with water temperature. In 2020, however, *R. mediterranea* was not observed in the same lakes, although temperature ranges were similar to previous studies. The fragmentary occurrence of the species in both countries does not provide clear evidence of the species’ environmental optima.

In addition to nutrient concentrations and temperature, intraspecific diversity and naturally occurring ecotypes should be included among factors determining the distribution of *R. raciborskii* in eastern Europe. A recent study on the global occurrence of *R. raciborskii* ecotypes indicated a few major ecotypes with unclear distribution (Baxter et al., 2022). This may indirectly explain the distribution pattern we observed: the expansion of ecotypes that inhabit western regions with milder climate was hampered in eastern regions with more severe climate. Only some strains could establish and sustain small populations under these conditions.

## 5 Conclusion

Although our study showed increasing prevalence of *R. raciborskii* in the eastern regions of Poland, expansion into this region appears to be constrained by the more severe climate conditions. Although *R. raciborskii* occurred in several new lakes, biomass was similar across all lakes, the maximum contribution to total phytoplankton biomass in western regions of Poland was higher than observed in the 2016 study. Air temperature was again a significant factor determining biomass. As such, the contribution of *R. raciborskii* to total phytoplankton biomass was much lower in cooler eastern regions than warmer western regions with longer vegetation season. In addition to air temperature, nutrient concentrations played a major role as *R. raciborskii* occurred more frequently in lakes with low TRP and high TN concentrations than more eutrophic lakes. Catchment area land use, both agricultural and urban, had no direct effect on the occurrence or biomass of *R. raciborskii*. However, these catchment types should not be neglected when considering non-point sources of nutrients. In addition to environmental variables, the role of ecotypes at the edge of species geographical range should be considered when explaining range expansion. *R. mediterranea* was only detected in small number of lakes in Poland without a clear distributional pattern. In Lithuania, it was present in only one lake, compared to previous years when it was more widely distributed. In both countries, *R. mediterranea* biomass was low and therefore relationships with environmental factors could not be quantified. In sum, these results highlight the importance of climate variables for the distribution of invasive cyanobacteria.

## Data Availability

The original contributions presented in the study are included in the article/Supplementary material, further inquiries can be directed to the corresponding author.
